# Wm. R. Warner & Co.

**Published:** 1882-02-20

**Authors:** 


### EDITORIALS AND MISCELLANEOUS.

THE splendid house of Wm. R. Warner & Co., has a new advertisement in this issue. Don't fail to read it.

